# Precontoured Clavicular Locking Plate with Broad Lateral End: A Newly Designed Plate for Lateral Third Clavicle Fractures

**DOI:** 10.5704/MOJ.1803.003

**Published:** 2018-03

**Authors:** KC Kapil-Mani, P Acharya, S Arun

**Affiliations:** Department of Orthopaedics, Civil Service Hospital, Kathmandu, Nepal

**Keywords:** broad lateral end, complications, functional outcomes, lateral third clavicle fractures, precontoured locking plates

## Abstract

**Introduction:** Various treatment modalities are available but no consensus has been reached for optimal treatment of lateral third clavicle fractures. Precontoured locking plates with broad lateral end for multiple screws fixation is a newly designed plate for lateral third clavicle fractures. The objective of our study was to analyse the functional outcomes as well as complications of this technique in a significant number of cases with long follow-up duration.

**Materials and Methods:** Forty-six patients with distal third clavicle fractures were treated by precontoured clavicular locking plate with broad lateral end. Functional outcomes were assessed on the basis of Constant-Murley Shoulder Outcome Score and University of California, Los Angeles (UCLA) Shoulder Rating Score, active shoulder range of motion, time for fracture union and coraco-clavicular distance.

**Results:** The mean Constant-Murley score was 92.56±4.47 (range: 79-98) for injured side and 96.22±2.23 (range: 90-100) for normal side with p-Value 0.56. Mean coraco-clavicular distance at final follow-up was 10.52±1.13 mm (range 9.7 to 11.7 mm) in injured side and 10.25±0.98 mm (range 9.6 to 11.2 mm) in normal side. Mean UCLA Shoulder Rating Score was 32.55±2.12 (range: 27-34) for injured side and 33.46±1.88 (range: 31- 35) on normal side with p value 0.58. No major complications that necessitated revision of surgery occurred in our study.

**Conclusion:** This newly designed plate seemed extremely useful in successful union of lateral third clavicle fractures, with reduced rate of complications like fixation failures, iatrogenic rotator cuff injury, AC joint osteoarthritis and sub-acromial bursitis, with good functional outcomes.

## Introduction

Lateral end clavicle fractures account for 12 to 15% of clavicle fractures^1^. Rate of nonunion in these fractures can be as high as 22 to 44% because of strong displacing force between weight of the arm distracting the distal fragment and counter-pull of trapezius on the proximal fragment^2,3,4^. Restricted shoulder movement, persistent pain, loss of endurance and strength of shoulder may develop in case the fractures did not unite properly^5^. Treatment of established nonunion in lateral third clavicle fractures remains a surgical challenge to even experienced orthopaedic surgeon^6,7^. Various treatment modalities are available but no consensus has been reached for optimal treatment of these fractures^8^. Different treatment options are trans-articular or extra-articular Kirschner wire fixation^9^, Knowles pin fixation^10^, tension band wire fixation^3,11^,^12^ and coracoclavicular screw fixation^13^. However, these fixation methods are associated with various complications like pin migration, degeneration of acromio-clavicular joint, loss of reduction, penetration of screw through the bone and skin ulceration because of pin irritation^9,11^. Furthermore, rigid fixation and early mobilization is difficult after the above mentioned methods because of small comminuted soft meta-physeal distal bony fragment which does not provide the stable fixation construct^12^. Some authors introduce the hooked plate with an extension under the acromion to provide more stable fixation of fragments regardless of bone quality^14,15^. However, these plates are associated with rotator cuff injury, sub-acromial impingement or bursitis, clavicular stress fracture at medial end of plate and need for early removal of plate^16,17^.

Recently, precontoured clavicular locking compression plate with broad lateral end has been introduced that enables stable angular fixation of distal fragment regardless of bone quality, reduces the risk of loss of reduction, requires minimal bending of plate and does not lead to iatrogenic rotator cuff injury or sub-acromial impingement.

The purpose of our study was to evaluate prospectively clinical and radiological outcomes with use of precontoured locking plate with broad lateral end for the treatment of displaced lateral third clavicle fractures.

## Materials and Methods

**Fig. 1:**
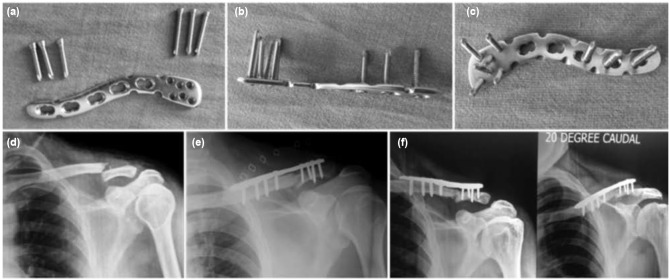
(a) Pre-contoured clavicular locking plate with broad lateral end along with small and large sized screws from superior surface, (b) from lateral surface, (c) from ventral surface, (d) Antero-posterior (AP) radiograph of shoulder joint showing lateral third clavicle fracture, (e) Immediate post-operative radiograph after fixation with locking plate and (f) AP and 20 degrees caudal view of clavicle 9 months after surgery showing union of fracture.

This was level IV prospective analytical study performed in Civil Service Hospital, Kathmandu, Nepal, from November 2011 to October 2016. Approval was obtained from the institutional review board of our hospital. Fifty patients with distal third clavicle fractures were treated by precontoured clavicular locking plate with broad lateral end (Fig. 1a-1c). Functional outcomes and time to union of the fracture were assessed at least one year after surgery. Four patients missed out on the follow-up visit and the remaining 46 patients were included in the study. Neer’s type II unilateral clavicular fractures with normal shoulder function were included in the study while those with acromio-clavicular (AC) joint dislocation, previous surgery on shoulder joint, pathological fractures and hemiparesis were excluded from the study. All surgery were performed by the first and second authors either independently or jointly.

**Fig. 2:**
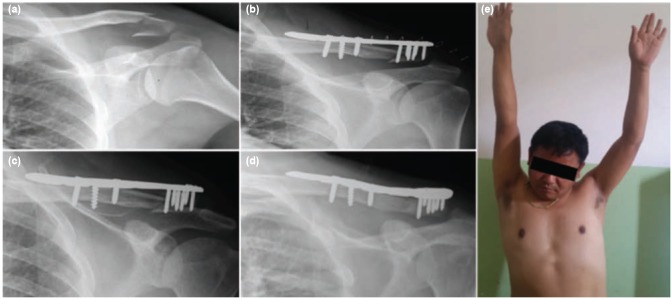
(a) Antero-posterior radiograph of shoulder joint showing comminuted lateral third clavicle fractures, (b) Immediate postoperative radiograph after fixation with pre-contoured lateral third clavicular plate, (c) Radiograph of clavicle 6 weeks after surgery, (d) Radiograph of clavicle 20 degrees caudal AP view 5 months after surgery showing bony union and (e) Abduction of shoulder joint 6 weeks after surgery.

**Fig. 3:**
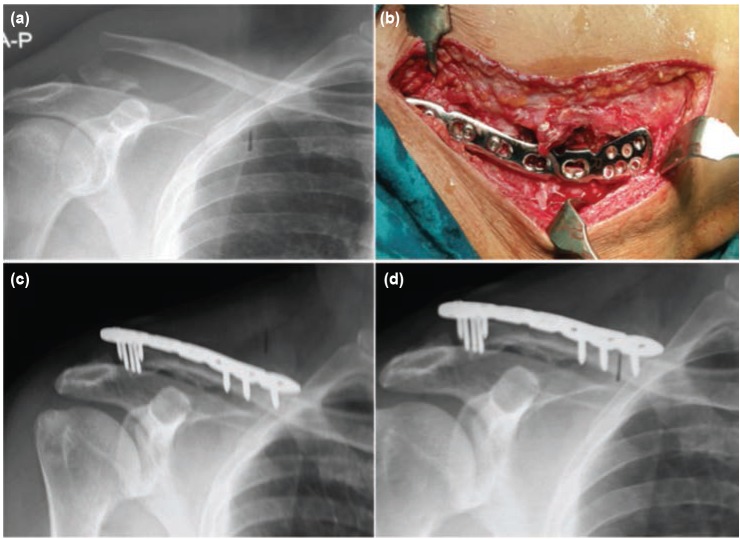
(a) Radiographs antero-posterior view of shoulder showing lateral end of clavicle fracture, (b) Intra-operative photograph after fixation with pre-contoured lateral third clavicular plate. Radiographs taken at (c) 6 months and (d) 9 months after surgery showing fracture union.

All the surgeries were performed either under general anaesthesia or inter-scalene block, with elevated head end of operating table. A curved incision was given over the lateral aspect of the clavicle. The deep tissue dissection was performed between the deltoid muscle anteriorly and trapezius muscle posteriorly to reach the fracture site. Exact location of the AC joint was identified with the use of Kirschner wire (K-wire) by locating the soft gap between lateral end of clavicle and acromion process. The AC joint capsule was left intact. The periosteum on the superior surface of clavicle was stripped from both the fracture fragments. The fracture was reduced with bone holding forceps and temporarily held with 2 mm K-wire passed through the acromion process. Now precontoured clavicular plate with broad lateral end for multiple screws was placed over the bone (Fig. 1a-1c). The plate was fixed with three to four cortical screws of size 3.5 mm in diameter on the medial end and four to six mini screws of size 2.3 mm in diameter on the lateral end (Fig 2a-2d and Fig 3a-3d). The position of screws on the lateral end of clavicle was confirmed by fluoroscopy and any intra-articular screws were removed. After fixation with the screws the provisional K-wire passed through the acromion was as a rule removed except in some cases it was retained to enhance the stability of fracture. Romovac drain was inserted and soft tissue was closed in layers.

Arm pouch sling was used for support of the clavicle in the immediate post-operative period. Pendulum exercise was started two to three days after surgery when pain had subsided. Arc of pendulum swing was gradually increased to 90 degree up to four weeks after surgery, after which the arm pouch sling was removed and active range of shoulder motion was started to achieve normal shoulder motion within two months (Fig 2e). Patients were followed up in outpatient department at two weeks, six weeks, three months, six months, one year and yearly after that. In each visit radiograph with antero-posterior view of shoulder joint was done to evaluate fracture union. Functional outcomes were assessed on the basis of Constant-Murley Shoulder Outcome Score and University of California, Los Angeles (UCLA) shoulder rating score, active shoulder ROM, time to union of the fracture and coraco-clavicular distance at the last follow-up for both the injured and normal shoulder joint (at least one year after surgery). Functional outcomes based on these scores were assessed during each visit. However, the final results were documented at last follow-up. The Constant-Murley Shoulder outcome score contains maximum score of 100 points with pain (15 points maximum), activities of daily living (20 points maximum), shoulder ROM (40 points maximum), and muscle power (25 points maximum). The UCLA shoulder rating score which has a maximum score of 35 points includes pain and function (each with ten points maximum), muscle power (active forward flexion), shoulder ROM (active forward flexion), and patient satisfaction, each with a maximum score of five points.

Statistical analysis was performed using the SPSS software version 16.0. Quantitative variables were documented as mean ± standard deviation. Constant-Murley score, UCLA shoulder rating score, and the coraco-clavicular distance between injured and normal shoulder were compared using the student t-test. P values <0.05 were considered statistically significant.

## Results

The average age of patients in our study was 32.44±7.75 years (range 18 to 57 years). There were 29 (63.04%) male and 17 (36.96%) female. Twenty five (54.35%) fractures were on the left side and 21 (45.65%) fractures on the right. Average time interval between the initial injury and surgery was 4.87±1.57 days (range: 2 to 7 days). The average time to union of the fracture was 15.28±2.45 weeks (range: 12 to 22 weeks) and mean follow-up duration was 33.68±11.52 months (range: 12 to 56 months). The average operating time was 65.47±8.87 minutes (range: 45 to 95 minutes). Mean Constant-Murley score, UCLA shoulder rating score and the coraco-clavicular distance between injured and normal shoulder are in Table I. Mean time for the patient to regain maximum range of motion, UCLA score and Constant-Murley shoulder score were determined one year after surgery. Mean active range of motion of shoulder joint was abduction 158±5.56 degrees (range: 135-170 degrees); forward flexion 164.73±4.64 degrees (range: 138-170 degrees); external rotation 47.76±3.48 degrees (range: 36 to 55 degrees) and internal rotation 68.85±5.34 degrees (range: 55 to 80 degrees). No major complications that necessitated revision of surgery occurred in our study. There were some minor complications like one case each of superficial wound infection, intra-articular placement of screw, plate back out, three cases of transient stiffness of shoulder, four cases of paresthesia over the shoulder joint.

**Table I:** Constant-Murley Score, coraco-clavicular distance, UCLA Shoulder Rating Score in normal and injured sides

**Parameters**	**Normal side**	**Injured side**	**Injured side**	**Injured side**	**p-Value**
		**(6 months)**	**(1 year)**	**(latest follow-up)**	
Mean Constant-Murley	96.22±2.23	92.56±4.47	94.46±3.42	94.68±3.23	0.56
score	(range 90-100)	(range 79-98)	(range 82-98)	(range 83-99)	
Mean coraco-clavicular	10.13±0.92 mm	10.25±0.98 mm	10.34±0.96 mm	10.52±1.13 mm	_
distance in immediate post-operative period	(range 9.5-11.1 mm)	(range 9.6-11.2 mm)	(range 9.5-11.4 mm)	(range 9.7-11.7 mm)	
Mean UCLA shoulder	33.46±1.88	30.29±2.08	32.28±2.11	32.55±2.12	0.58
rating score	(range 31-35)	(range 26-33)	(range 26-34)	(range 27-34)	

## Discussion

It is difficult to achieve stable fixation and early mobilization with previously proposed treatment methods in cases fractures of the lateral third of clavicle where the distal fragment is small and deforming forces are great^18-20^. No consensus has been established regarding the optimal operative treatment for these fractures^1^. Various methods of surgical treatment are available at present like-K wire fixation, tension band wiring, screw fixation, locking and non-locking plates^1^. We have used pre-contoured locking compression plate with broad lateral end for multiple screws which provides stable fixation of a small sized lateral fragment, allows early mobilization and secures bony union. This plate is anatomically designed to fit the contour of the lateral third of clavicle. It allows application of screws in different directions in the lateral third of clavicle and provides the multi-planar fixation and greater stability for small unstable lateral fragment. There are as many as six smaller locking screw holes available in the lateral end of this plate in different direction. Screws have increased pull-out strength and need not be engaged in the opposite cortex because of their locking mechanism. This design of the plate neutralizes the forces acting upon the fracture fragments and maintains strong hold even in osteoporosis fractures. So, it is extremely useful to achieve successful union of lateral third of clavicle fractures^8^. Stable fixation and early post-operative shoulder motion can be achieved even in osteoporotic bone without involving the acromio-clavicular (AC) joint or sub-acromial space. There are less complications like fixation failures, iatrogenic rotator cuff injury, AC joint osteoarthritis and sub-acromial bursitis. Stress fracture of clavicle is insignificant in this technique compared with hook plate fixation because of lack of stress riser at its medial end. Furthermore, it is not compulsory to remove the plate because of its low profile configuration and relatively bulky muscles at the lateral end of clavicle^1,8^.

The concept of locking plate fixation in the distal clavicle was first described by Kalamaras *et al*^3^ using distal radius locking plate. They concluded that use of locking plate gave superior results in the treatment of lateral third clavicle fractures as it had better control in the small distal fragment. Rieser *et al*^21^ performed the biomechanical analysis of various treatment modalities for distal clavicle fractures and reported that locking plate fixation had stable fixation biomechanically and provided superior clinical outcomes.

In our study, all the fractures united uneventfully. Functional outcomes based on the Constant-Murley score and UCLA shoulder rating scores were excellent and comparable to the normal side. No major complications that necessitated revision surgery occurred in our study except some minor complications like one case each of superficial wound infection, intra-articular placement of screw, plate back out, three cases of transient stiffness of shoulder and four cases of paresthesia over the shoulder joint. The results of our study are quite promising and supported well by few other studies in the literature. There were no cases with malunion or nonunion in our study. Likewise, other studies did not report non-union of lateral third clavicle fractures fixed with this plate. Paresthesia over the shoulder joint was found in slightly higher number of patients, accounting for 8.7% of cases in our study. Other studies in literature did not mention the exact percentage of paresthesia over the anterior aspect of shoulder joint after surgery.

Anderson *et al*^22^ reported 13 patients of lateral clavicle fractures fixed with locking compression plates and concluded that this technique was quite superior with high union rates, excellent functional outcomes and low complications rates. Klein *et al* compared the functional outcomes between the precontoured locking plates with suture augmentation in 16 patients and clavicular hook plates in 22 patients with lateral clavicular fractures. They found early fracture union in both group and those with precontoured locking plates resulted with the superior functional results^23^. The average time interval between the initial injury and surgery in our study was 4.87±1.57 days (range 2 to 7 days). We operated on all fractures relatively in the early stage and found good functional results. Chunline *et al* performed a comparative study between the locking compression plates in 36 patients and hook plates in 30 patients and found that functional outcomes were better in the group of locking plates^24^.

The review article of Sambandam *et al* concluded that clinical outcomes were equal in clavicular fractures fixed by flexible fixation like K-wires and tension banding wiring and rigid fixation by locking plates. However, they also noted that it was easy to remove the flexible implants under local anaesthesia while rigid implants required general anaesthesia for their removal^7^. Very few literatures were published regarding this fractures fixed with broad lateral end clavicular plates. Our study included a significant number of cases (46 patients) and functional outcomes were assessed in detail based on the Constant-Murley Score, UCLA Shoulder Rating Score and coraco-clavicular distance with long follow-up duration as compared to the previous studies. Mean time for patients with lateral third clavicle fractures fixed by this technique to get maximum range of motion, UCLA score and mean Constant-Murley Score was calculated one year after surgery. Mean Constant-Murley Score and UCLA Rating Score on both injured and normal side were nearly equal at final follow-up; however, not statistically significant with p-Value 0.56 and 0.58 respectively.

Because of the low profile character of plates, it is not always necessary to remove the plates. We did not remove any plates fixed for lateral third clavicle fractures and did not encounter any complications because of the plates. Because of muscle bulk in lateral third clavicle fractures, minor plate back-out does not cause much problems with resulting prominence of plates; however, significant amount of plate back-out causing prominence of plates needs removal of implants. Similarly, intra-articular placement of screws in some cases would need removal of offending screw later under image intensification guidance. Hence, it is noted that the lateral end clavicle plate was biomechanically stable and would give good results in the fracture fixation of the small distal fragments of the Neer's type II fractures. If magnetic resonance imaging (MRI) of injured side was needed, MRI can be performed after removal of plate in selected cases.

## Conclusion

Precontoured clavicular locking compression plate with broad lateral end with multiple screw holes allows application of screws in different directions in the lateral third of clavicle and provides the multi-planar fixation and greater stability for small unstable lateral fragment. So, it is extremely useful to achieve successful union of fractures of the lateral third of clavicle with reduced rate of complications like fixation failures, iatrogenic rotator cuff injury, AC joint osteoarthritis and sub-acromial bursitis with good functional outcomes.

## Conflict of Interest

The authors declare no conflict of interest.
